# Temporal-Spatial Pattern of Carbon Stocks in Forest Ecosystems in Shaanxi, Northwest China

**DOI:** 10.1371/journal.pone.0137452

**Published:** 2015-09-09

**Authors:** Gaoyang Cui, Yunming Chen, Yang Cao

**Affiliations:** 1 Institute of Soil and Water Conservation, Chinese Academy of Sciences and Ministry of Water Resources, Yangling, Shaanxi, China; 2 State Key Laboratory of Soil Erosion and Dryland Farming on the Loess Plateau, Northwest A and F University, Yangling, Shaanxi, China; DOE Pacific Northwest National Laboratory, UNITED STATES

## Abstract

The precise and accurate quantitative evaluation of the temporal and spatial pattern of carbon (C) storage in forest ecosystems is critical for understanding the role of forests in the global terrestrial C cycle and is essential for formulating forest management policies to combat climate change. In this study, we examined the C dynamics of forest ecosystems in Shaanxi, northwest China, based on four forest inventories (1989–1993, 1994–1998, 1999–2003, and 2004–2008) and field-sampling measurements (2012). The results indicate that the total C storage of forest ecosystems in Shaanxi increased by approximately 29.3%, from 611.72 Tg in 1993 to 790.75 Tg in 2008, partially as a result of ecological restoration projects. The spatial pattern of C storage in forest ecosystems mainly exhibited a latitude-zonal distribution across the province, increasing from north (high latitude) to south (low latitude) generally, which signifies the effect of environmental conditions, chiefly water and heat related factors, on forest growth and C sequestration. In addition, different data sources and estimation methods had a significant effect on the results obtained, with the C stocks in 2008 being considerably overestimated (864.55 Tg) and slightly underestimated (778.07 Tg) when measured using the mean C density method and integrated method, respectively. Overall, our results demonstrated that the forest ecosystem in Shaanxi acted as a C sink over the last few decades. However, further studies should be carried out with a focus on adaption of plants to environmental factors along with forest management for vegetation restoration to maximize the C sequestration potential and to better cope with climate change.

## Introduction

Forests play a key role in mitigating global climate change through the sequestration of the dominant greenhouse gas, carbon dioxide (CO_2_), which is induced by fossil fuel combustion and land use change [1]. It was estimated that 86% and 73% of the above- and below-ground C are globally stored in forests, respectively [2]. The precise and accurate quantitative evaluation of forest C retention and C stocks is fundamental to understanding the forest ecosystem C cycle and formulating policy related to forest conservation and management practices [3, 4].

Most studies on forest C sequestration have been conducted at national and regional scales, which provide knowledge on the contributions of forests as C sinks in the global C cycle [4–11]. However, a great deal of uncertainty remains regarding the C sink of forests with respect to magnitude and location due to the incomplete knowledge of the spatial and temporal dynamics of forest C storage. For example, estimates of the C sink in American forests from boreal to temperate and tropical biomes differ by more than 30-fold, ranging from 0.067 to 1.7 Pg C year^-1^ [8, 12]. For European forests, estimates vary from 0.068 Pg C year^−1^ to 0.7 Pg C year^−1^ [13]. The large discrepancies in estimates of forest C sinks, except for inherent geographic regional differences, can be ascribed to the different data sources, models or methods adopted within these studies. The data used by Fan et al. (1998) were atmospheric and oceanic CO_2_ data and model estimations, whereas land use change data were adopted by Houghton et al. (2000). Generally, land use change and forest inventories, originating from systematic and continuous monitoring at a large scale, are the most reliable data used to estimate the C sequestration of forests and to assess the function of forests as C sinks [10, 14, 15]. Although many studies of forest C sequestration using forest inventories at a large scale have achieved more reliable results [9, 10], many problems persist. First, a C concentration coefficient of 0.5 [9, 16] or 0.45 [17] was typically adopted to estimate the C storage of forests. Second, C storage in the understory, litter, and soil was not taken into account [18, 19]. These two deficiencies will ultimately result in an inaccurate and incomplete evaluation of forests as C sinks and hence necessitate more thorough research on the sequestration ability of forest ecosystems, including vegetation, litter, and soil at the small to medium scale.

Several provinces, such as Guangdong, Hainan, and Jilin, have launched such studies on comprehensive forest ecosystem C sequestration in China [20–22]; however, there is no integrated report for Shaanxi Province, which contains the most abundant forest resources in northwest China, although several inventory-based estimations of forest tree C with large variations have been conducted [23–25]. This deficiency poses an obstacle to an exact understanding of the role of forest ecosystems in the C budget and of whether forests are a sink or source of C in Shaanxi. In addition, there is no report on the temporal and spatial patterns of C storage in forest ecosystems in Shaanxi. Therefore, our objective is to examine the temporal and spatial patterns of C storage in forest ecosystems in Shaanxi over the period 1993–2008 to accurately evaluate the function of the forest C sink in Shaanxi. Specifically, we focus on 1) the variation of C density and storage in forest ecosystems in Shaanxi Province from 1993 to 2008, 2) the spatial distribution of C storage in Shaanxi, mainly through examining forest C stocks of cities from north to south with different climate conditions and, 3) the influence of different methods applied to the same database on estimates of C storage for forest ecosystems.

## Materials and Methods

### Ethics statement

Our study was based on forest inventory data and field sampling measurement. All necessary permits were obtained from Shaanxi bureau of forestry, and no endangered or protected species were involved in our field sampling.

### Description of Shaanxi Province

Shaanxi Province, located in northwest China, covers an area of 20.58×10^4^ km^2^, ranging from 105°29′ to 111°15′ E and from 31°42′ to 39°35′ N. The climate varies greatly in the region, spanning temperate, warm temperate, and north subtropical climatic zones from north to south, with a mean annual temperature of 8 to 16°C and mean annual precipitation of 320 to 1400 mm. The terrain of Shaanxi is complex and diverse, dominated by the Loess Plateau in the north, the Guanzhong Plain centrally, and mountains in the south, which occupy 45%, 19%, and 36% of the total territory, respectively. Temperate grassland, warm temperate deciduous broad-leaved forest, and subtropical evergreen broad-leaved forest are the main zonal vegetation types in Shaanxi. With its large area located on the Loess Plateau, Shaanxi has initiated many ecological restoration projects to control soil and water loss and for environmental improvement, such as the “Grain for Green” program, a project launched in the 1990s to convert croplands with slopes greater than 15° into woodland and grassland. Many tree species such as *Robinia pseudoacacia*, *Pinus tabulaeformis*, *Cunninghamia lanceolata*, *Cupressus funebris* and other drought-tolerant and soil-controled species were planted during the project [26]. In addition, another one project, “Natural Forest Resources Protection”, aiming to protect remaining natural forests with nursery of forbidden mountain forest such as *Abies fabri*, *Picea asperata*, and *Quercus spp*., was launched by Shaanxi government in 1998 [24]. The restoration of forest vegetation is a product of these efforts. According to forestry resource inventory data (2004), forest coverage in Shaanxi Province is 37.26% and is mainly distributed in the Qinling, Ba, Qiao, Guan, and Huanglong mountains, which account for 94% of the total stand volume [25].

### Data sets

#### Forest inventory database

The forest inventory database of Shaanxi Province was used to analyse the temporal and spatial patterns of C storage in forest ecosystems in this province. The continual inventory system of forest resources in Shaanxi was established in 1978 with the intersections of each grid used as reference points to establish a 28.28 m×28.28 m plot within a 4 km×8 km grid (6440 plots in total). Forest area and timber volume for each age class and forest type were inventoried in these plots once every five years, including information on the geographic locations of the plots, the names of dominant species, average tree height, average tree diameter at breast height (DBH), number of standing trees and 80 other factors. Four inventory periods, including 1989–1993, 1994–1998, 1999–2003, and 2004–2008, were used in this study.

#### Field investigation data

Because the forest inventory database only contains information on trees that could be used to estimate C storage, we also conducted field sampling to investigate C storage in the understory (shrub, herb), litter, and soil in 2012. According to forest types distribution map of Shaanxi and field survey with the assistance of local forestry bureau, a total of 121 field sampling sites of 16 forest types were located and selected from across Shaanxi Province proportional to the area or volume of the forest types from the latest inventory of Shaanxi. The allocation of sampling sites in each forest type was as follows: 1 for *Larix gmelinii*, 13 for *Pinus tabuliformis*, 4 for *Pinus armandii*, 3 for *Pinus massoniana*, 1 for other pines and conifer forests, 2 for *Cupressus funebris*, 55 for *Quercus* spp., 8 for *Betula* spp., 5 for hardwood forest, 13 for *Populus* spp., 1 for softwood forest, 8 for mixed broad-leaf forest, and 7 for mixed coniferous and broad-leaf forest. Three duplicate plots with 20 m apart from each other were set at each sampling site. The area of each plot was 20 m×30 m, and the same variables were measured in each plot as were measured in the forest inventory. In addition, plant tissue in trees, the understory, litter and soil were sampled to obtain a measurement of the C concentration using the potassium dichromate oxidation method in the laboratory [27]. Briefly, samples (2 g), dried and sieved, were oxidized by the mixed solution of K_2_Cr_2_O_7_ and H_2_SO_4_, with the remaining K_2_Cr_2_O_7_ subsequently deoxidized with solution of FeSO_4_. The C concentration in samples was obtained by the difference of Cr^6+^ before and after the oxidation of carbonaceous matter.

### Estimation of forest C storage

The forest ecosystem C storage consisted of C storage in tree, understory, litter and soil. We estimated the tree C based on forestry inventory dataset. And the relationships between C storage in trees and in the understory, litter, and soil were derived from field sampling for each forest type to estimate the C storage in the understory, litter, and soil of corresponding forest types during the four inventory periods. Though the relationship varied among years, we assumed that it did not change [21]. The relationships between C storage in understory, litter, soil layer, and tree layer derived from the 121 sites that belonged to coniferous forest types were applied to estimate C storage in understory, litter, and soil layers for *Abies* and *Picea*, *Cunninghamia lanceolata*, and *Tsuga chinensis* stands because there were no corresponding plots for these three forest types due to access limitations.

#### Estimation of tree C storage based on field observations

We measured the tree height and DBH of each tree in the plot and calculated the biomass of all components (leaves, branches, stem, roots, and bark) of each tree using allometric equations from the corresponding tree species. These components were also sampled to enable the measurement of the C concentration in the laboratory. Through multiplying the biomass of each component with the C concentration of the corresponding component of every tree and summing these values, we could obtain the C storage in the tree layer of every plot. The allometric equations for each tree species in Shaanxi Province were collected from previous studies (S1 Table); if there was not an allometric equation developed for a tree species, an equation of a similar species was selected as a substitute.

#### Understory and litter C estimation based on field observations

Three quadrats along diagonal per plot for both the shrub and herb layers were established to estimate the C storage. The shrub quadrats were 2 m×2 m, and the herb quadrats were 1 m×1 m. All individual plants in each quadrat were collected and weighed, and 30% of the collected materials from each quadrat were dried to measure the moisture content and determine the C concentration using the potassium dichromate oxidation method [27]. The C storage in the understory layer was estimated by multiplying the dry biomass of the shrubs and herbs from each quadrat converted using the moisture content and the corresponding C concentration.

Similar to estimates for the understory, three 1 m×1 m quadrats were also established in each plot for estimating the C storage in the litter layer. The analysis and calculation procedures were the same as those in the understory layer.

#### Estimation of soil C storage based on field observations

To determine C storage in the soil layer, three soil cores were collected with a soil auger (4.2 cm in diameter and 100 cm in depth) in each plot. We separated the soil core into five sections, 0–10, 10–20, 20–30, 30–50, 50–100 cm. Soil bulk density was measured within these five corresponding soil layers of every 1 m soil profile. The mixed soil samples of 3 cores from the same depth layer were processed using the potassium dichromate oxidation method to determine the organic matter content [27].

C storage in the soil layer of each plot was calculated using the following equation:

$$C_{SOCi}=\sum_{j=1}^{5}\left[\frac{1}{10}SOC_{\text{c}ij}\times BD_{ij}\times Depth_{ij}\right]$$ (1)

where *C*_*soci*_ is the C storage of soil in the *i*-th plot (Mg ha^-1^); *SOC*_*cij*_, *BD*_*ij*_, and *Depth*_*ij*_ are the C concentration, soil bulk density, and soil depth of the *j*-th soil layer in the *i*-th plot (j = 1, 2, 3, 4, 5 representing 0–10, 10–20, 20–30, 30–50, 50–100 cm, respectively), with the units of g kg^-1^, g cm^-3^, and cm, respectively; and 1/10 is the unit conversion factor.

#### Estimation of tree C storage based on forest inventory

The biomass in the tree layer of the forest inventory was estimated using biomass-volume relationships [28, 29].

B=aV+b (2)

where *B* and *V* are the forest stand biomass (Mg ha^-1^) and stand volume (m^3^ ha^-1^, for measurement method see reference [29]), respectively, and *a* and *b* are conversion parameters of a specific forest type (Table 1).

**Table 1:** Biomass-volume conversion formula for each forest type.

Forest type	Conversion formula	n	R^2^	Reference
*Abies and Picea*	B = 0.4642V+47.499	13	0.98	Xu et al., 1996 [28]
*Tsuga chinensis*	B = 0.4158V+41.3318	21	0.88	Fang et al., 1998 [29]
*Larix gmelinii*	B = 0.967V+5.7598	8	0.98	Xu et al., 1996 [28]
*Pinus tabuliformis*	B = 0.7554V+5.0928	82	0.96	Xu et al., 1996[28]
*Pinus armandii*	B = 0.5856V+18.7435	9	0.90	Fang et al., 2001 [9]
*Pinus massoniana*	B = 0.52V	12	0.92	Xu et al., 1996 [28]
Other pines and conifer forests	B = 0.5168V+33.2378	16	0.94	Fang et al., 2001 [9]
*Cunninghamia lanceolata*	B = 0.3999V+22.541	56	0.95	Fang et al., 2001 [9]
*Cupressus funebris*	B = 0.6129V+26.1451	11	0.96	Xu et al., 1996 [28]
*Quercus* spp.	B = 1.3288V-3.8999	3	1.00	Fang et al., 2001 [9]
*Betula* spp.	B = 0.9644V+0.8485	4	0.95	Xu et al., 1996 [28]
Hardwood	B = 0.7564V+8.3103	11	0.97	Xu et al., 1996 [28]
*Populus* spp.	B = 0.4754V+30.6034	10	0.86	Fang et al., 2001 [9]
Softwood	B = 1.0357V+8.0591	21	0.83	Fang et al., 2001 [9]
Mixed broad-leaf forest	B = 0.6255V+91.0013	19	0.86	Xu et al., 1996 [28]
Mixed coniferous and broad-leaf forest	B = 0.8019V+12.2799	9	0.99	Fang et al., 2001 [9]

B and V are the forest stand biomass (Mg ha^-1^) and stand volume (m^3^ ha^-1^). All the regression models are significant (P<0.05); Hardwood: wood density>0.7, which denotes that the hardness of the end of the wood is greater than 700 kg/ cm^2^; softwood: wood density<0.7.

The C storage in the tree layer for different forest types was calculated as follows:

$$SOC_{i}=\sum_{j=1}^{5}\left[\left(a_{i}V_{i}{}_{j}+b_{i}\right)\times Area_{i}{}_{j}\times Cconcentration_{i}\right]$$ (3)

where *i* is the forest type (*i* = 1–16, there are 16 forest types as described above); *j* is the age class of each forest type (*j* = 1–5, representing young, middle-aged, near-mature, mature, and over-mature forest, respectively; the age range of each class varied with forest types, more details please refer to technical guideline of Northwest Institute of Forest Investigation and Planning in S2 Table); *SOC*_*i*_ is the C storage in the tree layer of the *i*-th forest type (Mg); *V*_*ij*_ (m^3^ ha^-1^) and *Area*_*ij*_ (ha) are the forest stand volume and forest area, respectively, of *j*-th age class in the *i*-th forest type; and *a*_*i*_, *b*_*i*_, and *Cconcentration*_*i*_ are the conversion factors and C concentration, respectively, of *i*-th forest type. The C concentration for each forest type was measured during our field sampling plots in 2012.

Because the forest inventory was organized at a local city scale and then aggregated into a final database for the entire province, we calculated the C storage for each forest type at local city and whole province scales, respectively.

### Spatial distribution of forest C storage

Based on C storage in the tree layer, estimated from the forest inventory (2004–2008), and C storage in the understory, litter, and soil layers, estimated by the relationship between measurements of the shrub, herb, litter, and soil layer C and the tree layer C derived from field sampling plots in 2012, C storage of forest ecosystems for all 10 cities in Shaanxi Province were estimated for spatial distribution analysis.

### Influence of estimation methods on forest C storage

For comparison, two other methods were used in this study to estimate the C storage of forest ecosystems in Shaanxi Province over the period 2004–2008. The mean C density method calculated the C storage of each forest type by multiplying the mean ecosystem C density, obtained only from field sampling plots, by the forest area. The other method was an integrated approach that estimated tree layer C storage based on the forest inventory and estimated the C storage in the understory, litter, and soil layers by multiplying the mean C density of these layers by the area of field sampling. Because no field sampling site was established for *Abies* and *Picea*, *C*. *lanceolata*, and *T*. *chinensis*, the mean C density of the tree, understory, litter, and soil layers for these three forest types were calculated by averaging all the plots belonging to coniferous forest types. Hereafter, we refer to the methods described here as mean C density method and integration method, respectively, and the method introduced in previous sections as correlation method (Table 2).

**Table 2:** Methods used for estimation of forest ecosystem C stocks in our study.

Methods	Description
Correlation	Estimated tree layer C storage based on the forest inventory and estimated C storage in the shrub, herb, litter, and soil layers using the relationship between the tree layer and these layers derived from field observations
Mean C density	Calculated the C storage of each forest type by multiplying the mean ecosystem C density, obtained only from field observations in S4 Table, by the forest area.
Integration	Estimated tree layer C storage based on the forest inventory and estimated the C storage in the understory, litter, and soil layers by multiplying the mean C density of these layers in S4 Table with the area.

### Uncertainty analysis

During the estimation of the mean C density in tree, understory, litter, and soil layers and the total ecosystem based on field sampling plots, uncertainties were unavoidable. The uncertainty was addressed at three levels: the uncertainties of each C pool in the ecosystem (including the C density of tree, understory, litter, and soil layers); the uncertainties of ecosystem C density; and the uncertainties in up-scaling C storage to the province level [30].

The 95% confidence interval (2SE) is typically used to assess the uncertainty in component C density [31], where SE is the standard error of the mean. To assess the uncertainty for ecosystem C density, a simple error propagation method, summing the square of each component’s uncertainty and then determining the square root of the sum based on probability theory, was used. The uncertainty for the C storage of each forest type was calculated by multiplying the uncertainty of each ecosystem by the area of the ecosystem because there was no uncertainty regarding the area [32], and we used a similar method to calculate ecosystem uncertainty to estimate the uncertainty of total C storage in forest ecosystems in Shaanxi Province. All data analysis was performed using procedures of SPSS 16.0 (SPSS Inc., USA) and the accepted significance level was α = 0.05.

## Results

### Forest cover and type during 1949–2008

**Fig 1:**
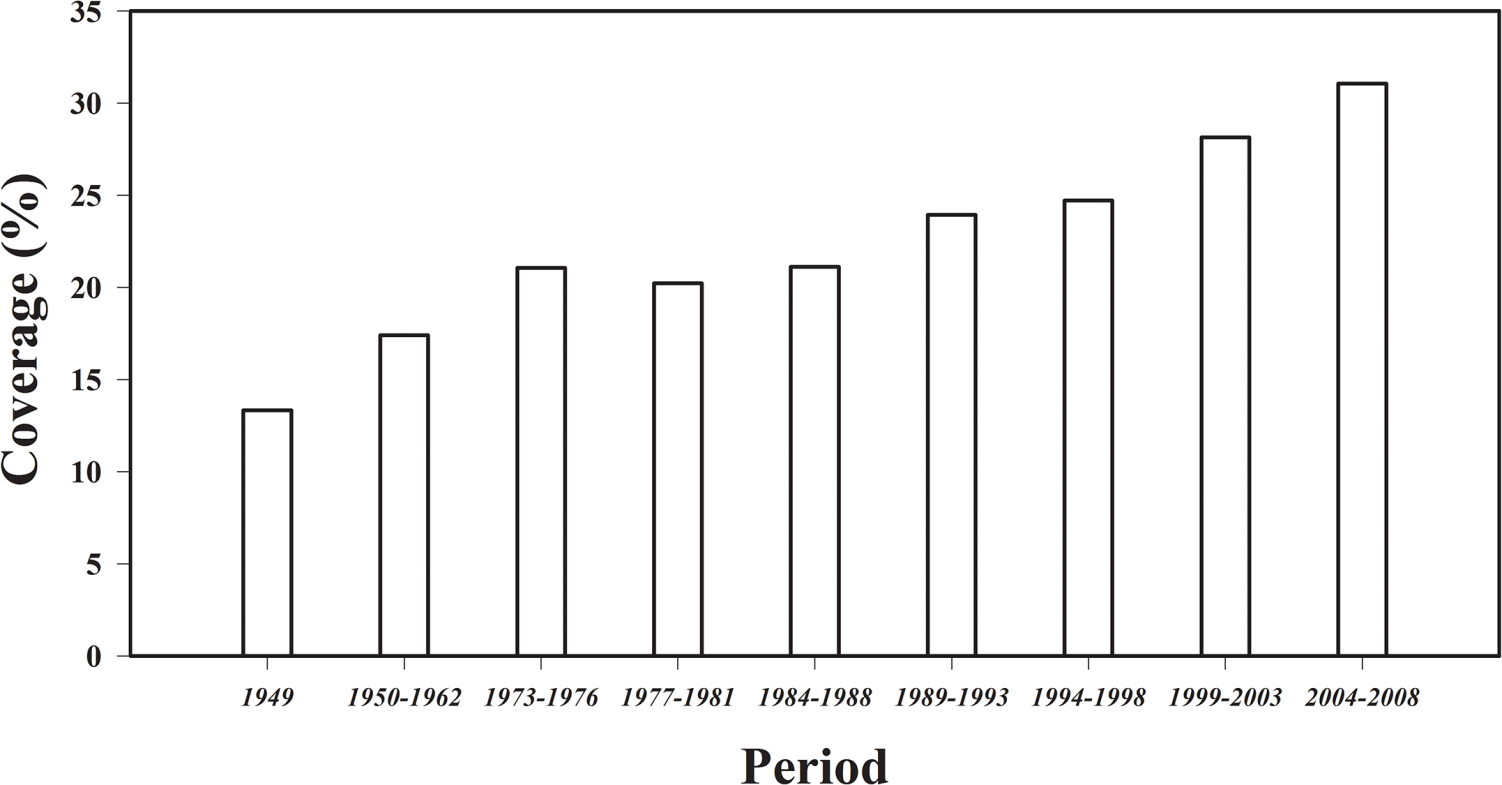
Change in the forest coverage in Shaanxi from 1949 to 2008. The data from 1949 to1988 was from Fang and Chen (2001) [23].

Forest cover in Shaanxi Province increased from 13.3% in 1949 to 31.1% in 2008, during which there were two periods of relatively rapid increase, 1949–1976 and 1998–2008 (Fig 1). There were 13 different forest types during the period of 1989–1993 and 1994–1998, which increased to 16 in 1999–2003 and 2003–2008 due to the addition of other pines and conifer forests, mixed broad-leaf forest, and mixed coniferous and broad-leaf forest types (S3 Table). Among these forest types, *Quercus* spp. and hardwood forest were the forest types with the largest proportions within the total forest area.

### Forest C density and storage from 1993 to 2008

The average C density of forest ecosystems in Shaanxi Province was 124.19±4.29, 121.94±4.60, 120.82±7.40, and 123.68±5.77 Mg ha^-1^ during the four periods, respectively (Table 3). However, the ecosystem C density of most forest types increased, with the exceptions of *Quercus* spp., *Betula* spp., and softwood, during 1993–2008 (Table 3). The C density varied within forest types from 76.4 Mg ha^-1^ in *C*. *lanceolata* in 1989–1993 to 173.1 Mg ha^-1^ in the mixed broad-leaf forest in 2004–2008. The C density was lower in *P*. *massoniana* and *C*. *lanceolata* among the four periods; whereas it was higher in *Abies* and *Picea*, *T*. *chinensis*, and the mixed broad-leaf forest during the latter two periods and in *L*. *gmelinii* and *Betula* spp. during the former two periods.

**Table 3:** C density, storage, and area of forest ecosystems in Shaanxi Province during four periods: 1989–1993, 1994–1998, 1999–2003, and 2004–2008 (mean±95%CI).

	1989–1993			1994–1998			1999–2003			2004–2008		
Forest type	Area (100 ha)	Density (Mg ha^-1^)	Storage (Tg)	Area (100 ha)	Density (Mg ha^-1^)	Storage (Tg)	Area (100 ha)	Density (Mg ha^-1^)	Storage (Tg)	Area (100 ha)	Density (Mg ha^-1^)	Storage (Tg)
*Abies and Picea*	512	126.05±12.90	6.45	384	165.15±14.95	6.34	416	163.44±16.30	6.80	448	160.78±13.85	7.20
*Tsuga chinensis*	160	109.69±11.01	1.75	160	147.70±11.33	2.36	160	163.40±16.46	2.61	192	166.07±14.82	3.19
*Larix gmelinii*	160	135.56±45.66	2.17	160	144.97±39.63	2.32	192	136.66±54.71	2.62	128	136.33±43.38	1.75
*Pinus tabuliformis*	4510	95.53±9.01	43.09	4831	99.42±7.94	48.03	5180	104.35±11.02	54.05	5533	108.61±11.62	60.09
*Pinus armandii*	1152	94.61±38.63	10.90	1247	115.33±44.28	14.38	1343	121.09±50.58	16.26	1215	122.26±52.98	14.85
*Pinus massoniana*	1088	83.26±10.53	9.06	1183	86.42±9.16	10.22	1471	89.98±11.17	13.24	1471	96.80±14.57	14.24
Other pines and conifer forests							64	116.79±34.27	0.75	96	111.81±32.63	1.07
*Cunninghamia lanceolata*	544	76.42±8.31	4.16	576	102.56±10.96	5.91	640	106.90±12.36	6.84	1024	109.32±12.20	11.19
*Cupressus funebris*	960	87.41±15.71	8.39	1023	108.57±24.22	11.11	1087	109.32±30.36	11.88	1536	106.70±29.10	16.39
*Quercus* spp.	19248	140.44±5.70	270.32	19648	126.97±6.24	249.47	24084	121.77±9.38	293.26	26420	132.22±9.96	349.32
*Betula* spp.	2080	150.61±15.41	31.33	2175	137.04±14.21	29.81	1920	132.45±18.66	25.43	2208	144.20±16.31	31.84
Hardwood	10171	119.41±15.69	121.45	11194	120.37±15.81	134.75	11606	121.13±29.60	140.59	14483	112.81±15.55	163.38
*Populus* spp.	3872	93.22±9.57	36.09	3166	116.44±9.99	36.86	2078	114.69±11.75	23.83	2587	118.59±13.39	30.68
Softwood	4799	138.69±5.58	66.56	5118	134.23±6.17	68.70	6360	128.34±6.21	81.62	5658	124.55±8.54	70.47
Mixed broad-leaf forest							768	168.96±15.91	12.98	671	173.08±13.26	11.61
Mixed coniferous and broad-leaf forest							320	133.52±15.15	4.27	256	135.29±18.48	3.46
Total	49256	124.19±4.29*	611.72	50865	121.94±4.60*	620.26	57689	120.82±7.40*	697.04	63926	123.69±5.77*	790.75

* These values represent the area-weighted mean C density of forest ecosystems during different periods.

The total C storage of forest ecosystems in Shaanxi Province increased from 611.7Tg in 1993 to 790.7 Tg in 2008, for a total growth of 29.3% over 15 years (Table 3). The C storage of most forest ecosystems generally increased during that period, whereas the growth trend ceased in the last period of 2004–2008 in *L*. *gmelinii*, *P*. *armandii*, and the softwood forest. *Quercus* spp. and the hardwood forest made up a large proportion of the total C storage, accounting for 391.8 (64.0%), 384.2 (61.9%), 433.9 (62.2%), and 512.7 (64.8%) Tg during the four periods, respectively (Table 3).

**Fig 2:**
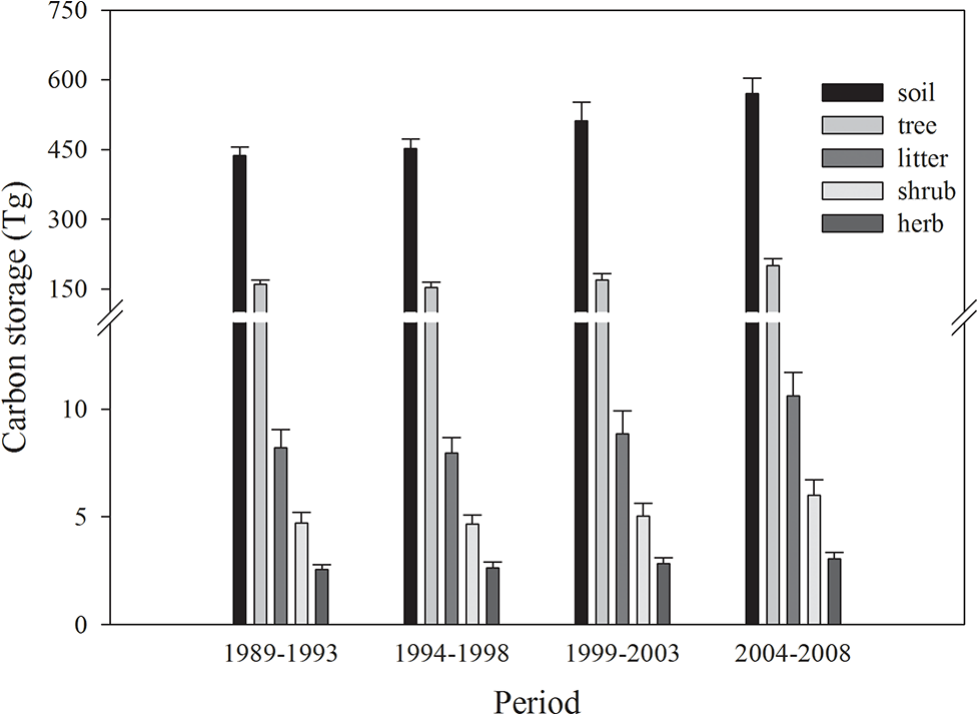
Carbon storage in soil, tree, shrub, herb, and litter layers of forest ecosystems in Shaanxi during 1989–1993, 1994–1998, 1999–2003, and 2004–2008.

The allocation of C stored in each layer of forest ecosystems was uneven among these four periods. The soil layer was the largest C pool within each ecosystem, accounting for more than 70% of the total storage in forest ecosystems during each period (S3 Table). Moreover, forest and soil layers stored almost the entire C stock of the forest ecosystem, with 596.2 (97.4%), 605.0 (97.5%), 680.3 (97.6%), and 771.1 (97.5%) Tg during the four periods, respectively (Fig 2). The C storage increased in all the five layers from 1993 to 2008, though a slight decrease occurred in the period of 1994–1998 for tree, shrub, and litter layers.

### Spatial distribution of forest C storage during 2004–2008

The C storage of forest ecosystems was distributed heterogeneously across Shaanxi Province. The C storage was highest in the southwest and the north, mainly including Hanzhong (180.2 Tg), Ankang (141.1 Tg), and Yan’an (159.6 Tg), where the Qinling, Ba, Guan, Huanglong, and Qiao mountains are located. The C storage of forest ecosystems was in the middle of the range in west central (Baoji, Xi’an) and east central (Weinan, Shangluo) locations, with the lowest C storage in other regions (Table 4).

**Table 4:** Forest ecosystem C storage of each city in three regions of Shaanxi Province during 2004–2008.

Region	Range	MAP(mm)	MAT(°C)	City and C storage(Tg)
North	N 35°35′01″-39°35′07″	400–600	7–11	Yulin(34.6), Yan’an (159.6)
	E 107°14′51″-111°14′31″			
Central	N 33°50′34″-35°35′01″	500–700	11–13	Xi’an(46.2), Baoji(86.6), Xianyang(39.1), Weinan(40.1), Tongchuan(13.1)
	E 106°18′25″-110°36′36″			
South	N 31°42′24″-33°50′34″	700–900	14–15	Hanzhong(180.1), Ankang(141.1), Shangluo(102.2)
	E 105°29′14″-111°01′54″			

MAP: mean annual precipitation, MAT: mean annual temperature.

### Forest C storage estimated by different methods

As noted above, the total C storage of forest ecosystems in Shaanxi Province in 2004–2008 was 790.7 Tg using Method 1, which is considerably lower than the estimate from Method 2 (864.5 Tg) and slightly higher than the estimate from Method 3 (778.1 Tg) (Table 5).

**Table 5:** Total C storage of forest ecosystems using three methods during 2004–2008 in Shaanxi Province.

		C density (Mg ha^-1^)			Total C storage (Tg)		
Forest type	Area (100 ha)	Ecosystem	Tree	shrub-soil	Method 1	Method 2	Method 3
*Abies and Picea*	448	139.62	59.27	90.25	7.20	6.25	6.70
*Tsuga chinensis*	192	139.62	63.49	90.25	3.19	2.68	2.95
*Larix gmelinii*	128	192.33	41.34	139.96	1.75	2.46	2.32
*Pinus tabuliformis*	5533	132.71	20.47	87.49	60.09	73.43	59.74
*Pinus armandii*	1215	164.27	29.60	104.64	14.85	19.96	16.31
*Pinus massoniana*	1471	101.06	13.59	56.97	14.24	14.87	10.38
Other pines and conifer forests	96	178.49	23.83	104.49	1.07	1.71	1.23
*Cunninghamia lanceolata*	1024	139.62	21.41	90.25	11.19	14.30	11.43
*Cupressus funebris*	1536	100.36	19.67	67.67	16.39	15.41	13.42
*Quercus* spp.	26420	144.86	37.52	91.49	349.32	382.73	340.85
*Betula* spp.	2208	198.91	45.14	159.44	31.84	43.92	45.17
Hardwood	14483	107.37	23.28	78.36	163.38	155.51	147.21
*Populus* spp.	2587	137.82	27.57	97.04	30.68	35.65	32.23
Softwood	5658	143.37	32.21	95.88	70.47	81.12	72.48
Mixed broad-leaf forest	671	161.90	69.12	114.23	11.61	10.86	12.30
Mixed coniferous and broad-leaf forest	256	144.03	39.73	90.95	3.46	3.69	3.35
Total	63926				790.75	864.55	778.07

The C density of ecosystems, estimated by field sampling only, was the sum of the mean C density in tree, shrub, herb, litter, and soil layers. The C density of trees was estimated through a forest inventory, and shrub-soil was the sum of the mean C density in the shrub, herb, litter, and soil layers based on field sampling measurements. Method 1: a combination of forest inventory data (tree layer) with field sampling measurements (estimates of storage in the shrub, herb, litter, and soil layers using the relationship between the tree layer and these layers). Method 2: a mean C density method, which calculated the C storage for each forest type by multiplying the mean ecosystem C density by the area obtained only from field sampling. Method 3: an integrated method that estimated C storage in the tree layer based on the forest inventory and estimated C storage in the understory, litter, and soil layers by multiplying the mean C density of these layers by the area based on field sampling.

## Discussion

Many ecological restoration programs have been launched in Shaanxi Province since the 1950s due to the serious soil erosion throughout the province, especially on the Loess Plateau. Because of these efforts to improve the environment, forest coverage increased 2.2% per year from 1949 to 2008, especially during the periods of 1949–1976 and 1994–2008, with rates of 2.3% and 2.6% per year, respectively, primarily owing to three projects: extensive tree planting in the 1970s, the “Grain for Green” program and the “Natural Forest Resources Protection” project in 1998. The C storage of forest ecosystems in Shaanxi Province was also estimated based on four periodic forest inventories and field sampling measurements in our study. We found that the total C storage of forest ecosystems greatly increased by 179 Tg from 1993 to 2008, and the increase was especially obvious after the initiation of two major projects in 1998, with approximately 95% (170 Tg) of the total increase occurring from 1998–2008. Specifically, the “Grain for Green” program in Shaanxi has converted about 73.8×10^4^ ha croplands vulnerable to soil erosion into woodland with the afforestation of *P*. *tabuliformis*, *P*. *massoniana*, *C*. *lanceolata*, *Betula*, *Robinia pseudoacacia* and other plantations, and approximate 67.7×10^4^ ha natural secondary forest dominated by *Quercus* spp. was established under the Natural Forest Resources Protection project (Table 3). All of these two main projects resulted in an increase of about 160 Tg forest C stores contributed by each other equally. The C sinks of the forest ecosystem in Shaanxi Province illustrated the role played by forest ecosystems in the global C cycle. The C density, however, varied slightly and even declined from 124.19 Mg ha^-1^ in 1993 to 120.82 Mg ha^-1^ in 2003 because a large number of young plantations with low C density were planted within the project. For example, the plantations accounted for nearly one-quarter of the total forest area. As the trees grow, however, the C density will increase, as exemplified by the growth of C density from 2003–2008 and more C will be sequestered in the future in forest ecosystems in Shaanxi Province.

The soil C pool is an important component of C storage in forest ecosystems, and more than two-thirds of the total C in forest ecosystems is stored in forest soil worldwide [6], which was in agreement with our results (Fig 2). However, this pool was usually not included in previous studies [5, 9, 18] or was simply estimated using the ratio of soil C to vegetation biomass [4], resulting in an incomplete and inaccurate understanding of the C sink of forest ecosystems and greater uncertainty regarding the C sinks of terrestrial ecosystems. Here, regression formulas of C stocks between trees and the soil for main tree species were derived from direct field sampling measurements to estimate the C stocks in soil not included in the inventory (S1 Fig). The most derived equations indicated a significant (*P*<0.05) relationship between soil and tree C, although the large range in the determinate coefficient(R^2^) was from 0.09 to 0.53, which may be explained by other factors not included in our study that affected the C storage of soil, such as location, forest types, dominant species, and soil types [21]. As reported in previous studies, a lots of soil variables including organic C, total N, total exchangeable bases, K^+^, Mg^2+^, and clay were positively associated with tree biomass and had a profound effect on forest tree C stores [33]. Besides, slope, altitude and other topographic variables also influenced the aboveground biomass and were responsible for the variations [34], which implied that there existed complicated factors and mechanisms controlling the interaction between tree and soil. All of these would potentially result in a big uncertainty concerning the relationship and also a low R^2^ in our study. The ratio of soil C to vegetation biomass used to estimate soil C stocks, however, did not reflect the real interaction between the soil and trees, which varied with environmental factors [35], although it simplified the calculation procedure. In addition, another specific method was applied to estimate soil C in most European countries by either applying a constant ratio of soil C per hectare to total forest area or by applying ratios specific to soil type and soil type areas [36], which likely induced an overestimation, as discussed in the last paragraph. Despite the existence of many defects, the simple empirical model between soil C and trees, parameterized from our direct ecosystem studies, is an effective attempt to investigate the interaction between plants and soil. Further efforts are needed for a deeper understanding of this interaction with abiotic and biotic aspects taken into account. Moreover, the terrestrial soil, not just forest soil, represents a much larger pool, with its C stocks exceeding the sum of C in vegetation and the atmosphere [9]. A slight variation in soil C storage will induce drastic changes in atmospheric CO_2_ concentrations; thus, it is essential to focus on the C dynamics of terrestrial soil (including forest soil) in the future to better mitigate global climate change.

The uneven spatial distribution of C storage in forest ecosystems in Shaanxi Province reflected the effect of climatic conditions on vegetation growth and C accumulation. Overall, the C storage of forest ecosystems increased from north to south across the whole province (Table 4) as a result of improvements in heat and water conditions beneficial for vegetation growth and litter input, accompanied by increased precipitation and temperature. Yan’an, however, exhibited higher C storage in the north because the two main forest zones in the Huanglong and Qiao mountains are located there [25]. In addition, many ecological restoration projects contributed greatly to the increase in vegetation coverage in Yan’an [26]. In contrast, the city of Yulin had the lowest C stock, even though vegetation restoration programs have been on-going for a long period as in Yan’an. The contrasting results are mainly attributed to the fact that extreme drought restricted the growth and restoration of vegetation in Yulin, which lies in an arid steppe transition belt from typical grassland to a semi-desert landscape. Therefore, different forest management practices, such as tree species selection, should be applied to optimally adapt restoration and C sequestration projects to regional climate conditions.

Compared to the results of Zhou et al. (2000), who reported that the average C density of forest ecosystems in China was 258.83 Mg ha^-1^, with 193.55 Mg ha^-1^ in soil, 57.07 Mg ha^-1^ in vegetation, and 8.21 Mg ha^-1^ in the litter [37], the average C density in 2008 (123.68 Mg ha^-1^) was much lower in forest ecosystems in Shaanxi Province. This huge difference suggests that the quality of forest stands was poor and the forest was too young in Shaanxi Province, where more than 57.72% of forest area and 48.80% of total C storage was attributed to young forests, resulting in a considerably lower forest C density. However, the average C densities in the soil and vegetation layers of forest ecosystems in Shaanxi (S3 Table) were similar to those reported by Li (2002) and Wang et al. (2001), respectively [17, 38]. For example, Li (2002) reported that the average C density of forest soil in China was 81.39 Mg ha^-1^, and the mean C density of vegetation was 36–42 Mg ha^-1^ in the study of Wang et al. (2001). The distinct findings of the average C density in Chinese forest ecosystems between these studies reflects the uncertainty in the estimation of forest C density at a national scale based on the different methods and data sources employed, as demonstrated by different estimates of the C storage in forest ecosystems during 2004–2008 in our study (Table 5). Therefore, the data sources and the application of appropriate methods were crucial for an accurate estimation of C density and storage in forest ecosystems. These three methods represented different technologies used in previous studies. The mean C density method, a method similar to that recommended by the international biological program (IBP), calculates the total C storage by multiplying the mean forest C density with the forest area based on field sampling measurements, resulting in an overestimation due to the better growth conditions of selected plots compared to those of the entire forest area. However, this higher discrepancy was not universal. For example, the average C density in *Abies*, *Picea*, and *T*. *chinensis* estimated in field sampling sites was lower than that based on the forest inventory (S3 and S4 Tables) because these natural forests are distributed mainly in mountainous and remote regions, where direct measurements are difficult, leading to a bias in the distribution of sampling sites towards stands with younger ages and lower C densities. This finding is also applicable to other natural coniferous forests, from which the densities of these two forest types were calculated. The integrated method, however, produced a lower estimate of the total storage than correlation method, even though it applied a higher mean C density of understory, litter, and soil layers based on field measurements, with the exception of the storage in the tree layer, which was the same in both methods. The underestimation was chiefly attributed to the large difference in soil storage estimates between these two methods in addition to the relative lower C density in the understory and litter layers (S3 and S4 Tables). The C storage of soil in *P*. *massoniana*, *C*. *funebris*, *Quercus* spp., and the hardwood forest was largely calculated using correlation method as opposed to integration method, whereas the C storage of the soil in *Betula* spp. was estimated as smaller according to correlation method than according to integration method. The increase in the C storage in the soil for the four aforementioned forest types offset that in the last forest type due to their high proportion of forest area. In fact, the combined use of these three methods was a compromise technique, reflecting different methodologies used within certain periods. Further studies concerning the dynamics of ecosystem C and the overall C budget should adopt appropriate methods that best fit the data used in their research.

## Conclusion

Based on the forest inventory in addition to the field measurements, we studied the temporal and spatial patterns of C in forest ecosystems in Shaanxi Province. As the total C stocks increased from 611.72 Tg in 1993 to 790.75 Tg in 2008, forest ecosystems in Shaanxi Province acted as a net C sink, with a mean C uptake of 11.94 Tg per year in recent decades, which indicates that forests play an important role in the global C cycle and climate change mitigation. The C density, however, varied slightly and even declined from 124.19 Mg ha^-1^ in 1993 to 120.82 Mg ha^-1^ in 2003 as a result of a large number of young plantations with low C density planted within ecological restoration projects. Moreover, strong C sequestration potential will continue to manifest in forest ecosystems in Shaanxi Province following the growth of these young forests. The latitudinal-zonal distribution of forest ecosystem C storage reflects the effect of environmental conditions, chiefly water and heat factors, on forest growth and C sequestration. Forest management should focus plant adaptions to environmental constraints to achieve the maximum C sequestration potential in the future. The application of different data sources and methods, however, will result in uncertainty in estimations of C stocks in forest ecosystems; accurate estimations are essential to effectively evaluating the function of forest ecosystems as C sinks and should receive more attention in future studies concerning the C cycle.

## Supporting Information

S1 Fig Regression relationships between soil and tree C density derived from field sampling measurements for main tree species. (a), Mixed coniferous and broad-leaf forest; (b), *Pinus massoniana*; (c), *Pinus tabuliformis*; (d), *Cupressus funebris*; (e), *Betula* spp.; (f), *Quercus* spp.; (g), Hardwood; (h), *Populus* spp.; (i), Mixed broad-leaf forest.
(TIF)

S1 Table Allometric scaling equations for the biomass and C concentration (C%)^a^ of different components of forest species in Shaanxi. (DOC)

S2 Table The range of age class for main tree species*. (DOCX)

S3 Table C density, storage, area of forest ecosystems in Shaanxi province during four periods,1989–1993, 1994–198, 1999–2003, and 2004–2008. * The area-weighted mean C density in each layer of forest ecosystem during 2004–2008 in Shaanxi province; hardwood (wood density>0.7), softwood (wood density<0.7).
(DOCX)

S4 Table Mean C density of tree, shrub, herb, litter, soil layer, and ecosystem based on field sampling measurement. n is the number of plots used in calculating mean C density in each layer for 16 forest types; hardwood (wood density>0.7), softwood (wood density<0.7). ** the plots for these forest types were combination of *Larix gmelinii*, *Pinus tabuliformis*, *Pinus armandii*, *Pinus massoniana*, Other pines and conifer forests, with *Cupressus funebris*.
(DOCX)
