# Supplementary figures and images for: Temporal-Spatial Pattern of Carbon Stocks in Forest Ecosystems in Shaanxi, Northwest China
Source: PLoS One. 2015 Sep 9;10(9):e0137452. doi: 10.1371/journal.pone.0137452 (PMC4564278; doi:10.1371/journal.pone.0137452)

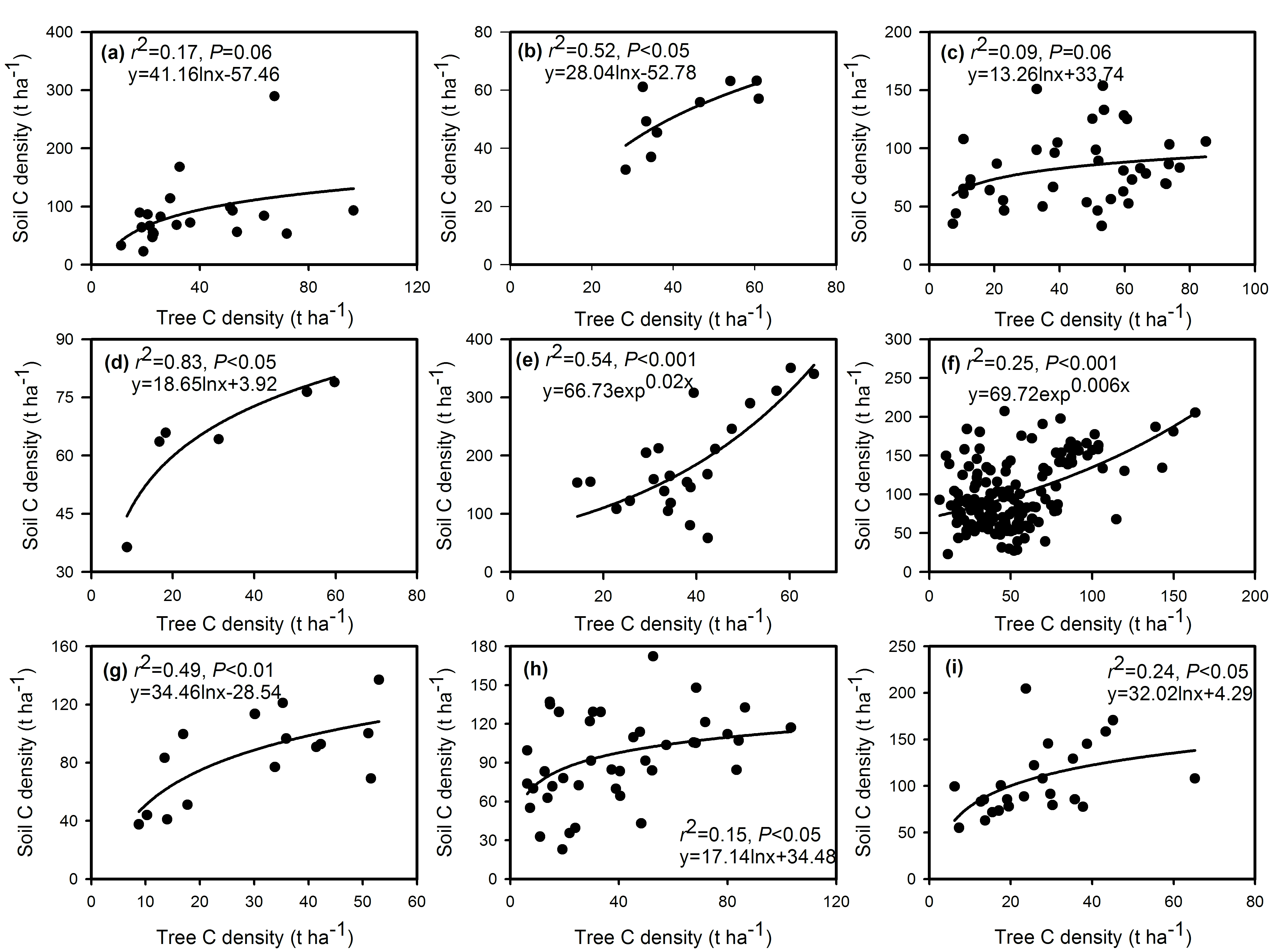

Supplement: S1 Fig — (a), Mixed coniferous and broad-leaf forest; (b), Pinus massoniana; (c), Pinus tabuliformis; (d), Cupressus funebris; (e), Betula spp.; (f), Quercus spp.; (g), Hardwood; (h), Populus spp.; (i), Mixed broad-leaf forest. (TIF) [file pone.0137452.s001.tif]
