# Supplementary material for: Temporal-Spatial Pattern of Carbon Stocks in Forest Ecosystems in Shaanxi, Northwest China
Source: PLoS One. 2015 Sep 9;10(9):e0137452. doi: 10.1371/journal.pone.0137452 (PMC4564278; doi:10.1371/journal.pone.0137452)
Supplement: S1 Table — (DOC) [file pone.0137452.s002.doc]

**Table S1.** Allometric scaling equations for the biomass and C concentration(C%)^a^ of different components of forest species in Shaanxi.

| Species/Composition | Allometric Scaling Equation | C (%) | Reference |
| --- | --- | --- | --- |
| *Larix gmelinii* |  |  |  |
| Stem | W = 0.01367 (D^2^H )^0.99794^ | 50.41 |  |
| Bark | W = 0.01072 (D^2^H )^0.80398^ | 49.39 |  |
| Branch | W =0.07802 D^2.04597^ | 52.34 |  |
| Root | W = 0.03136D^2.18625^ | 38.47 |  |
| Leaf | W =0.03184 (D^2^H )^1.90488^ | 52.57 |  |
| *Pinus tabuliformis* |  |  |  |
| Stem | W = 0.009741(D^2^H )^1.04086^ | 49.86 |  |
| Bark | W = 0.01072 (D^2^H )^0.80398^ | 50.42 |  |
| Branch | W = 0.0169 D^2.57733^ | 51.54 |  |
| Root | W = 0.015891D ^2.28692^ | 49.20 |  |
| Leaf | W = 0.00599 D ^2.57495^ | 51.47 |  |
| *Pinus armandii* |  |  |  |
| Stem | W = 0.01308(D^2^H )^1.0038^ | 50.11 |  |
| Bark | W = 0.01072 (D^2^H )^0.80398^ | 51.82 |  |
| Branch | W =0.0055 (D^2^H )^1.0439^ | 49.53 |  |
| Root | W =0.0033(D^2^H )^1.0148^ | 47.17 |  |
| Leaf | W = 0.0011(D^2^H )^1.12566^ | 49.61 |  |
| *Pinus massoniana* |  |  |  |
| Stem | W = 0.0459 (D^2^H )^0.8867^ | 58.60 |  |
| Bark | W = 0.01072 (D^2^H )^0.80398^ | 54.55 |  |
| Branch | W = 0.0127(D^2^H )^0.7886^ | 49.19 | This study* |
| Root | W = 0.0298 (D^2^H ) ^0.7415^ | 46.80 |  |
| Leaf | W = 0.0283(D^2^H ) ^0.6012^ | 49.53 |  |
| *Betula spp.* |  |  |  |
| Stem | W =0.02275 (D^2^H )^0.91035^ | 46.73 |  |
| Bark | W = 0.01388(D^2^H )^0.8102^ | 47.00 |  |
| Branch | W =0.002645D^3.35934^ | 48.38 |  |
| Root | W =0.01309 D ^2.6888^ | 44.81 |  |
| Leaf | W =0.003813D^2.3901^ | 49.07 |  |
| *Robinia pseudoacacia* |  |  |  |
| Stem | W =0.02583 (D^2^H)^0.6841^ | 46.64 |  |
| Branch | W =0.00464D^3.2181^ | 45.65 |  |
| Bark | W =0.00763(D^2^H)^0.0447^ | 44.68 |  |
| Leaf | W =0.0234D^1.9277^ | 48.22 |  |
| Root | W =0.01779D^2.6448^ | 39.59 |  |
| *Q. variabilis* |  |  |  |
| Stem | LnW = -3.7447+0.9679Ln(D^2^H) | 43.00 |  |
| Bark | LnW = -3.2565+0.7156Ln (D^2^H) | 42.23 |  |
| Branch | LnW = -4.8449+1.0013Ln( D^2^H) | 40.10 | Cheng et al, 2007 |
| Leaf | LnW= -3.3569+0.605 Ln( D^2^H) | 45.44 |  |
| Root | LnW= -2.9066+0.8144Ln( D^2^H) | 41.66 |  |
| *Q. wutaishanica* |  |  |  |
| Stem | LnW = 0.85136ln(D^2^H) -3.00984 | 43.84 |  |
| Branch | LnW=3.09503Ln(D)-5.31497 | 43.09 |  |
| Leaf | LnW=2.17397Ln(D)-3.98976 | 45.03 | Zhang,1990 |
| Root | LnW=1.79711Ln(D)-1.93175 | 38.65 |  |
| Bark | LnW=0.72629Ln(D2H)-3.39474 | 42.09 |  |

W, D, and H stand for biomass, diameter at breast height, and stem height, respectively. ^a^, the C concentration for each component was determined in our study. *The equations collected from our study was compiled though China website of carbon forest( http://www.carbontree.com.cn )

**References in Supplementary information**:

Zheng, BL., 1990. Study on biomass and productivity of *Quercus wutaishanica* stands in Ziwuling forest region of Shaanxi Province. Chinese Journal of Northwestern College of Forestry 5, 1­­­­­–7.

Cheng TR, Ma QY, Feng ZK, Luo X 2007 Research on forest biomass in Xiaolong Mountains, Gansu Province. Journal of Beijing Forestry University 29, 31-36.
