# Supplementary material for: Temporal-Spatial Pattern of Carbon Stocks in Forest Ecosystems in Shaanxi, Northwest China
Source: PLoS One. 2015 Sep 9;10(9):e0137452. doi: 10.1371/journal.pone.0137452 (PMC4564278; doi:10.1371/journal.pone.0137452)
Supplement: S2 Table — (DOCX) [file pone.0137452.s003.docx]

**Table S2.** The range of age class for main tree species*

| Species | Age class (year) | | | | |
| --- | --- | --- | --- | --- | --- |
|  | Young | Middle-aged | Near-mature | Mature | Over-mature |
| *Picea asperata*  *Tsuga chinensis*  *Cupressus funebris* | 0-60 | 61-100 | 101-120 | 121-160 | 161- |
| *P. tabulaeformis*  *P. armandii*  *P. massoniana* | 0-20 | 21-30 | 31-40 | 41-60 | 61- |
| *Abies fabri*  *Larix gmelinii* | 0-40 | 41-80 | 81-100 | 101-140 | 141- |
| Softwood | 0-10 | 11-15 | 16-20 | 21-30 | 31- |
| hardwood | 0-20 | 21-40 | 41-50 | 51-70 | 71- |
| *Cunninghamia lanceolata* | 0-10 | 11-20 | 21-25 | 26-35 | 35- |

*this is compiled from technical criteria of Northwest Institute of Forest Investigation and Planning (http://xby.forestry.gov.cn/.)
