# Supplementary material for: Temporal-Spatial Pattern of Carbon Stocks in Forest Ecosystems in Shaanxi, Northwest China
Source: PLoS One. 2015 Sep 9;10(9):e0137452. doi: 10.1371/journal.pone.0137452 (PMC4564278; doi:10.1371/journal.pone.0137452)
Supplement: S3 Table — * The area-weighted mean C density in each layer of forest ecosystem during 2004–2008 in Shaanxi province; hardwood (wood density>0.7), softwood (wood density<0.7). (DOCX) [file pone.0137452.s004.docx]

**Table S3** C density, storage, area of forest ecosystems in Shaanxi province during four periods,1989-1993, 1994-198, 1999-2003, and 2004-2008

| Year and forest type | Area (100 ha) | C density （Mg/ha） | | | | | | Total forest ecosystem C storage (Tg) |  |
| --- | --- | --- | --- | --- | --- | --- | --- | --- | --- |
|  |  | Tree layer | Shrub layer | Herb layer | Litter layer | Soil layer | Ecosystem |  |  |
| **1989—1993** | | | | | | | | | |
| *Abies and Picea* | 512 | 32.84 | 0.95 | 0.34 | 1.66 | 90.26 | 126.05 | 6.45 | |
| *Tsuga chinensis* | 160 | 21.65 | 0.63 | 0.23 | 1.09 | 86.09 | 109.69 | 1.75 | |
| *Larix gmelinii* | 160 | 40.75 | 0.45 | 0.43 | 1.33 | 92.61 | 135.56 | 2.17 | |
| *Pinus tabuliformis* | 4,510 | 12.95 | 0.33 | 0.16 | 1.19 | 80.91 | 95.53 | 43.09 | |
| *Pinus armandii* | 1,152 | 12.49 | 0.48 | 0.12 | 0.78 | 80.74 | 94.61 | 10.90 | |
| *Pinus massoniana* | 1,088 | 7.20 | 0.28 | 0.06 | 0.53 | 75.18 | 83.26 | 9.06 | |
| *Cunninghamia lanceolata* | 544 | 4.86 | 0.14 | 0.05 | 0.25 | 71.12 | 76.42 | 4.16 | |
| *Cupressus funebris* | 960 | 9.14 | 0.14 | 0.19 | 0.62 | 77.31 | 87.41 | 8.39 | |
| *Quercus* spp. | 19,248 | 43.70 | 1.16 | 0.33 | 2.15 | 93.10 | 140.44 | 270.32 | |
| *Betula* spp. | 2,080 | 49.93 | 2.02 | 0.96 | 3.35 | 94.35 | 150.61 | 31.33 | |
| Hardwood | 10,171 | 27.62 | 0.95 | 0.85 | 1.68 | 88.30 | 119.41 | 121.45 | |
| *Populus* spp. | 3,872 | 12.00 | 0.60 | 0.19 | 0.34 | 80.08 | 93.22 | 36.09 | |
| Softwood | 4,799 | 42.67 | 1.15 | 1.35 | 1.40 | 92.12 | 138.69 | 66.56 | |
| Total | 49,256 |  |  |  |  |  |  | 611.72 | |
| **1994—1998** |  | | | | | | | | |
| *Abies and Picea* | 384 | 62.75 | 1.82 | 0.65 | 3.17 | 96.75 | 165.15 | 6.34 | |
| *Tsuga chinensis* | 160 | 49.01 | 1.42 | 0.51 | 2.48 | 94.27 | 147.70 | 2.36 | |
| *Larix gmelinii* | 160 | 48.10 | 0.53 | 0.51 | 1.57 | 94.27 | 144.97 | 2.32 | |
| *Pinus tabuliformis* | 4,831 | 15.06 | 0.38 | 0.19 | 1.38 | 82.41 | 99.42 | 48.03 | |
| *Pinus armandii* | 1,247 | 24.91 | 0.96 | 0.24 | 1.55 | 87.66 | 115.33 | 14.38 | |
| *Pinus massoniana* | 1,183 | 8.52 | 0.33 | 0.08 | 0.62 | 76.87 | 86.42 | 10.22 | |
| **Table S3 continued** |  |  |  |  |  |  |  |  | |
| Period and forest type | Area (100 ha) | C density （Mg/ha） | | | | | | Total forest ecosystem C storage (Tg) | |
|  |  | Tree layer | Shrub layer | Herb layer | Litter layer | Soil layer | Ecosystem |  |  |
| *Cunninghamia lanceolata* | 576 | 17.21 | 0.50 | 0.18 | 0.87 | 83.80 | 102.56 | 5.91 | |
| *Cupressus funebris* | 1,023 | 20.84 | 0.32 | 0.44 | 1.41 | 85.56 | 108.57 | 11.11 | |
| *Quercus* spp. | 19,648 | 33.67 | 0.89 | 0.26 | 1.65 | 90.49 | 126.97 | 249.47 | |
| *Betula* spp. | 2,175 | 39.88 | 1.61 | 0.77 | 2.67 | 92.10 | 137.04 | 29.81 | |
| Hardwood | 11,194 | 28.28 | 0.98 | 0.87 | 1.72 | 88.54 | 120.37 | 134.75 | |
| *Populus* spp. | 3,166 | 26.10 | 1.30 | 0.42 | 0.74 | 87.87 | 116.44 | 36.86 | |
| Softwood | 5,118 | 39.33 | 1.06 | 1.24 | 1.29 | 91.31 | 134.23 | 68.70 | |
| Total | 50,865 |  |  |  |  |  |  | 620.26 | |
| **1999—2003** |  | | | | | | | | |
| *Abies and Picea* | 416 | 61.38 | 1.78 | 0.64 | 3.10 | 96.53 | 163.44 | 6.80 | |
| *Tsuga chinensis* | 160 | 61.36 | 1.78 | 0.64 | 3.10 | 96.52 | 163.40 | 2.61 | |
| *Larix gmelinii* | 192 | 41.59 | 0.46 | 0.44 | 1.35 | 92.81 | 136.66 | 2.62 | |
| *Pinus tabuliformis* | 5,180 | 17.89 | 0.45 | 0.22 | 1.64 | 84.14 | 104.35 | 54.05 | |
| *Pinus armandii* | 1,343 | 28.80 | 1.11 | 0.28 | 1.79 | 89.11 | 121.09 | 16.26 | |
| *Pinus massoniana* | 1,471 | 10.14 | 0.40 | 0.09 | 0.74 | 78.61 | 89.98 | 13.24 | |
| Other pines and conifer forests | 64 | 27.31 | 0.37 | 0.05 | 0.53 | 88.52 | 116.79 | 0.75 | |
| *Cunninghamia lanceolata* | 640 | 19.88 | 0.58 | 0.21 | 1.01 | 85.24 | 106.90 | 6.84 | |
| *Cupressus funebris* | 1,087 | 21.32 | 0.33 | 0.45 | 1.44 | 85.79 | 109.32 | 11.88 | |
| *Quercus* spp. | 24,084 | 29.95 | 0.79 | 0.23 | 1.47 | 89.32 | 121.77 | 293.26 | |
| *Betula* spp. | 1,920 | 36.58 | 1.48 | 0.71 | 2.45 | 91.23 | 132.45 | 25.43 | |
| Hardwood | 11,606 | 28.79 | 0.99 | 0.89 | 1.75 | 88.72 | 121.13 | 140.59 | |
| *Populus* spp. | 2,078 | 24.93 | 1.25 | 0.40 | 0.71 | 87.41 | 114.69 | 23.83 | |
| **Table S3 continued** |  |  |  |  |  |  |  |  | |
| Period and forest type | Area (100 ha) | C density （Mg/ha） | | | | | | Total forest ecosystem C storage (Tg) | |
|  |  | Tree layer | Shrub layer | Herb layer | Litter layer | Soil layer | Ecosystem |  |  |
| Softwood | 6,360 | 35.00 | 0.94 | 1.11 | 1.15 | 90.14 | 128.34 | 81.62 | |
| Mixed broad-leaf forest | 768 | 65.80 | 2.09 | 0.77 | 3.12 | 97.18 | 168.96 | 12.98 | |
| Mixed coniferous and broad-leaf forest | 320 | 38.42 | 0.97 | 0.29 | 1.95 | 91.89 | 133.52 | 4.27 | |
| Total | 57,689 |  |  |  |  |  |  | 697.04 | |
| **2004—2008** |  | | | | | | | | |
| *Abies and Picea* | 448 | 59.27 | 1.72 | 0.62 | 3.00 | 96.18 | 160.78 | 7.20 | |
| *Tsuga chinensis* | 192 | 63.49 | 1.84 | 0.66 | 3.21 | 96.87 | 166.07 | 3.19 | |
| *Larix gmelinii* | 128 | 41.34 | 0.46 | 0.43 | 1.35 | 92.75 | 136.33 | 1.75 | |
| *Pinus tabuliformis* | 5,533 | 20.47 | 0.52 | 0.25 | 1.88 | 85.49 | 108.61 | 60.09 | |
| *Pinus armandii* | 1,215 | 29.60 | 1.14 | 0.29 | 1.84 | 89.39 | 122.26 | 14.85 | |
| *Pinus massoniana* | 1,471 | 13.59 | 0.53 | 0.12 | 1.00 | 81.55 | 96.80 | 14.24 | |
| Other pines and conifer forests | 96 | 23.83 | 0.32 | 0.05 | 0.46 | 87.16 | 111.81 | 1.07 | |
| *Cunninghamia lanceolata* | 1,024 | 21.41 | 0.62 | 0.22 | 1.08 | 85.98 | 109.32 | 11.19 | |
| *Cupressus funebris* | 1,536 | 19.67 | 0.30 | 0.42 | 1.33 | 84.98 | 106.70 | 16.39 | |
| *Quercus* spp. | 26,420 | 37.52 | 1.00 | 0.28 | 1.84 | 91.58 | 132.22 | 349.32 | |
| *Betula* spp. | 2,208 | 45.14 | 1.83 | 0.87 | 3.03 | 93.34 | 144.20 | 31.84 | |
| Hardwood | 14,483 | 23.28 | 0.80 | 0.72 | 1.41 | 86.59 | 112.81 | 163.38 | |
| *Populus* spp. | 2,587 | 27.57 | 1.38 | 0.44 | 0.79 | 88.41 | 118.59 | 30.68 | |
| Softwood | 5,658 | 32.21 | 0.95 | 1.02 | 1.06 | 89.31 | 124.55 | 70.47 | |
| Mixed broad-leaf forest | 671 | 69.12 | 2.20 | 0.81 | 3.28 | 97.67 | 173.08 | 11.61 | |
| Mixed coniferous and broad-leaf forest | 256 | 39.73 | 1.01 | 0.30 | 2.02 | 92.22 | 135.29 | 3.46 | |
| Total | 63,926 | 31.38* | 0.94* | 0.48* | 1.66* | 89.24* |  | 790.75 | |

* The area-weighted mean C density in each layer of forest ecosystem during 2004-2008 in Shaanxi province; hardwood (wood density>0.7), softwood (wood density<0.7)
