# Supplementary material for: Temporal-Spatial Pattern of Carbon Stocks in Forest Ecosystems in Shaanxi, Northwest China
Source: PLoS One. 2015 Sep 9;10(9):e0137452. doi: 10.1371/journal.pone.0137452 (PMC4564278; doi:10.1371/journal.pone.0137452)
Supplement: S4 Table — n is the number of plots used in calculating mean C density in each layer for 16 forest types; hardwood (wood density>0.7), softwood (wood density<0.7). ** the plots for these forest types were combination of Larix gmelinii, Pinus tabuliformis, Pinus armandii, Pinus massoniana, Other pines and conifer forests, with Cupressus funebris. (DOCX) [file pone.0137452.s005.docx]

**Table S4** Mean C density of tree, shrub, herb, litter, soil layer, and ecosystem based on field sampling measurement

| Forest type | n | C density (Mg/ha) | | | | | | | | | | | |
| --- | --- | --- | --- | --- | --- | --- | --- | --- | --- | --- | --- | --- | --- |
|  |  | Tree layer | | Shrub layer | | Herb layer | | Litter layer | | Soil layer | | Ecosystem | |
|  |  | Mean | 2SE | Mean | 2SE | Mean | 2SE | Mean | 2SE | Mean | 2SE | Mean | 2SE |
| *Abies and Picea* | 72** | 49.36 | 5.67 | 1.33 | 0.26 | 0.52 | 0.14 | 3.69 | 0.70 | 84.71 | 10.69 | 139.62 | 13.36 |
| *Tsuga chinensis* | 72** | 49.36 | 5.67 | 1.33 | 0.26 | 0.52 | 0.14 | 3.69 | 0.70 | 84.71 | 10.69 | 139.62 | 13.36 |
| *Larix gmelinii* | 3 | 52.81 | 7.20 | 0.58 | 0.29 | 0.56 | 0.26 | 1.72 | 0.79 | 137.11 | 68.32 | 192.33 | 72.87 |
| *Pinus tabuliformis* | 39 | 45.27 | 7.84 | 1.15 | 0.30 | 0.56 | 0.21 | 4.15 | 0.95 | 81.63 | 9.65 | 132.71 | 14.14 |
| *Pinus armandii* | 12 | 59.62 | 13.69 | 2.31 | 0.79 | 0.58 | 0.25 | 3.70 | 1.79 | 98.05 | 40.05 | 164.27 | 43.18 |
| *Pinus massoniana* | 9 | 44.09 | 8.44 | 1.73 | 0.38 | 0.40 | 0.10 | 3.23 | 1.84 | 51.62 | 7.54 | 101.06 | 16.54 |
| Other pines and conifer forests | 3 | 74.01 | 25.02 | 1.01 | 0.39 | 0.15 | 0.04 | 1.43 | 0.57 | 101.90 | 33.73 | 178.49 | 56.38 |
| *Cunninghamia lanceolata* | 72** | 49.36 | 5.67 | 1.33 | 0.26 | 0.52 | 0.14 | 3.69 | 0.70 | 84.71 | 10.69 | 139.62 | 13.36 |
| *Cupressus funebris* | 6 | 32.98 | 20.34 | 0.50 | 0.07 | 0.70 | 0.55 | 2.23 | 1.18 | 64.24 | 15.11 | 100.36 | 34.75 |
| *Quercus* spp. | 165 | 53.37 | 4.54 | 1.42 | 0.25 | 0.41 | 0.06 | 2.62 | 0.31 | 87.05 | 5.87 | 144.86 | 7.29 |
| *Betula* spp. | 24 | 39.82 | 5.64 | 1.97 | 0.58 | 0.77 | 0.24 | 2.67 | 0.60 | 154.03 | 23.98 | 198.91 | 24.47 |
| Hardwood | 15 | 29.56 | 6.18 | 1.02 | 0.26 | 0.91 | 0.18 | 1.79 | 0.49 | 74.63 | 12.36 | 107.37 | 15.82 |
| *Populus* spp. | 39 | 41.51 | 8.34 | 2.07 | 0.72 | 0.67 | 0.17 | 1.18 | 0.36 | 93.11 | 10.84 | 137.82 | 15.84 |
| Softwood | 3 | 47.49 | 2.87 | 1.29 | 0.16 | 0.53 | 0.05 | 2.51 | 0.23 | 91.55 | 4.93 | 143.37 | 6.01 |
| Mixed broad-leaf forest | 24 | 47.83 | 5.65 | 1.65 | 0.34 | 0.56 | 0.10 | 2.30 | 0.40 | 109.72 | 11.57 | 161.90 | 13.69 |
| Mixed coniferous and broad-leaf forest | 21 | 53.10 | 6.88 | 1.58 | 0.40 | 0.49 | 0.10 | 3.00 | 0.45 | 85.88 | 11.86 | 144.03 | 13.69 |

n is the number of plots used in calculating mean C density in each layer for 16 forest types; hardwood (wood density>0.7), softwood (wood density<0.7)

** the plots for these forest types were combination of Larix gmelinii, Pinus tabuliformis, Pinus armandii, Pinus massoniana, Other pines and conifer forests, with Cupressus funebris
